# Trump 2 at war with science

**DOI:** 10.1590/0102-311XEN027025

**Published:** 2025-04-11

**Authors:** Kenneth Rochel de Camargo

**Affiliations:** 1 Instituto de Medicina Social Hesio Cordeiro, Universidade do Estado do Rio de Janeiro, Rio de Janeiro, Brasil.

Donald Trump’s second term - supported by an alliance of authoritarianism, religious fundamentalism, white supremacy, and ultra-neoliberalism - was met with apprehension by both the American and international scientific communities as soon as the election results were announced. The recent memory of his climate change denial, erratic policies during the COVID-19 pandemic, the bizarre attempt to manually alter a cyclone trajectory forecast map, and other similar actions during his first administration fueled the sense of alarm. And, as expected, the attack on science, even more intense than anticipated, is part of the blitzkrieg that the “new-old” administration unleashed against institutions and the legal foundations of the United States itself. Immediately, important scientific journals, such as *The Lancet* and the *British Medical Journal* (BMJ), published editorials sounding the alarm ^1^ ^,^ ^2^.

The attack on science takes place on several fronts. First, the determination to reduce the United States government workforce, as part of a policy to cut spending at any cost and without further criteria, is severely impacting several research agencies, such as the National Science Foundation (NSF) ^3^ ^,^ ^4^. The NSF is being forced to reduce its staff by up to 50% in the coming months, in addition to having its budget threatened with a two-thirds cut ^4^. Similar threats weigh on other vital research agencies, such as the National Oceanic and Atmospheric Administration (NOAA), the United States government’s official climate agency, and the National Aeronautics and Space Administration (NASA).

From a health perspective, the situation is even more worrying. In addition to being hit by the blind fury of cost-cutting no matter what, the nomination of Robert F. Kennedy Jr. as the new secretary of the Department of Health and Human Services (DHSS), equivalent to the position of Health Minister, is in and of itself worrying. RFK, as he is known, has been repeatedly characterized as a science denier, particularly anti-vaccine, and is part of a group of 12 individuals identified as the greatest disseminators of anti-vaccine information on the internet ^5^. An episode is particularly remarkable: after a critical mistake caused the deaths of two children following vaccination in Samoa (Polynesia), RFK’s interference further aggravated the crisis of trust in vaccines, leading to an even greater drop in vaccination coverage that led to the deaths of dozens of children in that archipelago-nation in the Pacific ^6^. RFK’s appointment as secretary had already been widely condemned, including by the American Public Health Association (APHA), in an open letter published on their website ^7^.

The DHSS oversees several institutions essential for health research, such as the National Institutes of Health (NIH) and the Centers for Disease Control and Prevention (CDC). One of Trump’s first acts was to halt any activity of these agencies, interrupting committees that would recommend funding proposals, essential conferences for health actions and research, staff hiring, and even scientific publication ^8^ and data dissemination ^9^. For the first time in 60 years, the publication of the *Morbidity and Mortality Weekly Report* (MMWR), an important epidemiological report, has been interrupted ^1^. The MMWR resumed its publication 15 days later, but apparently with significant gaps in tracking the ongoing avian influenza outbreak in the country.

Jay Bhattacharya, appointed president of the NIH, gained attention during the COVID-19 pandemic for being one of the signatories of the *Great Barrington Declaration*, a denialist document that criticized social distancing measures from an economic perspective and served as the basis for absurd proposals to delegate control of the pandemic to a supposed herd immunity, at a time when vaccines were still unavailable ^10^. The NIH is the largest funder of biomedical research in the United States, one of the largest, if not the largest in the world, and the expected interference from the new administration in its direction clouds future prospects of scientific development in health.

The determination to cut expenses at the NIH, in addition to reducing total amount for direct research funding, further compromises its development by setting a 15% limit on the so-called indirect costs. Universities and research institutes use these costs to cover expenses related to laboratories, equipment, and essential personnel for research infrastructure, which were previously at an average of 30%, and could be more than double that for major universities ^4^ ^,^ ^11^. 

Beyond appointing clearly unqualified individuals to leadership positions, restricting funds, and cutting staff, the current administration also seeks to censor scientists, prohibiting the funding of studies and the publication of articles by government officials containing references to words that have been included in an Inquisition-like “index prohibitorum”. Based on the ideological crusade against diversity, equity, and inclusion (DEI) initiatives, as well as any reference to gender -especially the very existence of transgender people -, entire governments’ webpages were deleted. Authors of articles previously submitted for publication demanded their return, and even previously allocated funding now faces an uncertain future ^8^ ^,^ ^12^.

Although opposing actors have risen recently, especially in the judicial sphere, in which several judges have issued rulings blocking some of the most egregious madness of this new administration’s early days, Trump controls the House of Representatives, the Senate, and even the Supreme Court until the next United States midterm elections, greatly limiting the reach of any resistance.

The prospects for scientific development in the U.S. in the coming years, particularly in the field of health, are bleak. Given the nation’s significant role in the international scientific landscape, the effects of these attacks will certainly be felt worldwide. Combined with equally reprehensible actions, such as withdrawal from the World Health Organization (WHO) and dismantling the United States Agency for International Development (USAID) - despite some of the latter’s questionable actions in the past - this projects a bleak outlook for global public health in the near future. The joint efforts of international agencies and the NIH were fundamental to face the pandemic, as it were during in the fight against HIV/AIDS. There is no indication that new global health challenges will not emerge in the coming years, especially given the worsening climate crisis - another field of research, now in the crosshairs of Trump 2’s demolition crews. 

The global scientific community must find alternatives to deal with the ongoing disaster in the United States, while also denouncing abuses and supporting affected scientists as much as possible. Brazil, in particular, should seek to diversify and strengthen multiple partnerships to minimize the inevitable negative impacts of a government openly at odds with science.
