# Supplementary material for: Trump 2 at war with science
Source: Cad Saude Publica. 2025 Apr 11;41(4):e00027025. doi: 10.1590/0102-311XEN027025 (PMC11996179; doi:10.1590/0102-311XEN027025)
Supplement: Translation

# Translation: Trump 2 em guerra contra a ciência

**DOI:** 10.1590/0102-311XPT027025

**Authors:** Kenneth Rochel de Camargo

**Affiliations:** 1 Instituto de Medicina Social Hesio Cordeiro, Universidade do Estado do Rio de Janeiro, Rio de Janeiro, Brasil.

A ascensão ao poder de Donald Trump pela segunda vez, amparado numa aliança de autoritarismo, fundamentalismo religioso, supremacismo branco e ultraneoliberalismo, foi vista com apreensão pela comunidade científica americana e internacional desde que os resultados da eleição foram anunciados. A memória recente do negacionismo climático, a política errática frente à pandemia de COVID-19, a bizarra tentativa de alterar à mão um mapa de previsão de trajetória de um ciclone e outros atos semelhantes na primeira administração de Trump justificavam esse sentimento de alarme. E, como era de se esperar, o ataque à ciência, em dimensão até maior do que se esperava, é parte da verdadeira *blitzkrieg* (guerra relâmpago) que a “nova-velha” administração desencadeou contra instituições e o próprio arcabouço legal daquele país. Imediatamente, importantes revistas científicas, como o *The Lancet* e o *British Medical Journal* (BMJ), publicaram editoriais soando o alarme ^1^ ^,^ ^2^.

O ataque à ciência ocorre em várias frentes. Em primeiro lugar, a determinação em reduzir a força de trabalho a serviço do governo americano, como parte de uma política de cortar gastos de qualquer modo e sem maior critério, atinge duramente vários órgãos ligados à pesquisa, como a Fundação Nacional de Ciência (NSF, acrônimo em inglês) ^3^ ^,^ ^4^, forçada a reduzir seu quantitativo de pessoal em até 50% nos próximos meses, além de ter seu orçamento ameaçado de redução em dois terços do valor anterior ^4^. Ameaças semelhantes pesam sobre outras agências relevantes para a pesquisa, como a Administração Oceânica e Atmosférica Nacional (NOAA, acrônimo em inglês), órgão climatológico oficial do governo americano, e a Administração Nacional de Aeronáutica e Espaço (NASA, acrônimo em inglês).

Do ponto de vista da área de saúde, o quadro é ainda mais preocupante. Além de ser também atingido pela fúria cega do corte de gastos a todo custo, a nomeação de Robert F. Kennedy Jr. como novo secretário do Departamento de Saúde e Serviços Humanos (DHSS, acrônimo em inglês), equivalente ao cargo de ministro da saúde, é, em si, alarmante. RFK, como é conhecido, já foi caracterizado repetidas vezes como negacionista da ciência, em especial antivacina, fazendo parte de um grupo de 12 indivíduos identificados como os maiores difusores de informações antivacina na internet ^5^. Um episódio em particular se destaca: depois de um erro crasso ter causado a morte de duas crianças após serem vacinadas, em Samoa (Polinésia), a interferência de RFK agravou ainda mais a crise de confiança nas vacinas, levando a uma queda ainda maior na cobertura vacinal, que levou à morte de dezenas de crianças na nação-arquipélago no Pacífico ^6^. A indicação de RFK para esse posto já havia sido amplamente denunciada, inclusive pela Associação Americana de Saúde Pública (APHA, acrônimo em inglês), em carta aberta publicada em seu site ^7^.

O DHSS abarca em seu âmbito diversas instituições essenciais para a pesquisa em saúde, como o Instituto Nacional de Saúde (NIH, acrônimo em inglês) e o Centros de Controle e Prevenção de Doenças (CDC, acrônimo em inglês). Uma das primeiras medidas de Trump foi congelar qualquer atividade desses órgãos, interrompendo comitês que indicariam propostas para financiamento, conferências essenciais para ações e pesquisa em saúde, a contratação de pessoal e mesmo a publicação científica ^8^ e a divulgação de dados ^9^, interrompendo, pela primeira vez em 60 anos, a publicação do *Morbidity and Mortality Weekly Report* (MMWR), importante informe epidemiológico ^1^. O MMWR foi restabelecido cerca de 15 dias depois, mas, aparentemente, com importantes lacunas no acompanhamento do surto de gripe aviária que se desenrola naquele país.

O indicado para a presidência do NIH, Jay Bhattacharya, destacou-se durante a pandemia de COVID-19 por ser um dos signatários da *Declaração de Great Barrington*, documento negacionista que criticava as medidas de distanciamento social com base numa ótica economicista e que serviu de base a propostas absurdas de delegar o controle da epidemia a uma suposta imunidade de rebanho em um momento em que ainda não se dispunha de vacinas ^10^. O NIH é o maior financiador de pesquisas biomédicas nos Estados Unidos, um dos maiores, senão o maior do mundo, e a interferência já prometida da nova administração nos seus rumos turva as perspectivas futuras de desenvolvimento científico nessa área.

A determinação do corte de gastos no NIH, além de reduzir o total de fundos para o financiamento direto de pesquisas, compromete ainda mais o seu desenvolvimento ao determinar um teto de 15% para os chamados custos indiretos, usados por universidades e institutos de pesquisa para custear gastos com laboratórios, equipamentos e pessoal indispensáveis à infraestrutura de pesquisa, que estavam num patamar médio de 30%, podendo ser mais que o dobro disso no caso de grandes universidades ^4^ ^,^ ^11^.

Para além de nomear pessoas claramente inadequadas para cargos de liderança, restringir fundos e cortar pessoal, a atual administração visa também exercer censura sobre cientistas, proibindo o financiamento de pesquisas e a publicação de artigos por funcionários do governo contendo alguma referência a palavras que foram incluídas num *index prohibitorum* digno da Inquisição. Tendo por base a cruzada ideológica voltada contra iniciativas de diversidade, equidade e inclusão (DEI), bem como qualquer referência à noção de gênero, em especial à própria existência de pessoas transgênero, páginas inteiras de sites do governo foram apagadas, autores de artigos já enviados para publicação demandaram a sua devolução às revistas para as quais haviam enviado e mesmo financiamentos já alocados têm futuro incerto ^8^ ^,^ ^12^.

Ainda que reações contrárias tenham se multiplicado nos últimos dias, sobretudo na esfera judicial, na qual vários juízes emitiram sentenças barrando alguns dos desvarios mais egrégios desse momento inicial do novo governo, é fato que, ao menos nos próximos dois anos, até as próximas eleições legislativas de meio termo nos Estados Unidos, Trump conta com o controle da Câmara, do Senado e mesmo da Suprema Corte, limitando muito o poder de alcance dessa resistência.

As perspectivas para o desenvolvimento da ciência nos Estados Unidos nos próximos anos, em particular na área da saúde, são sombrias. Dado o papel relevante que esta nação representa no cenário internacional da ciência, os efeitos desses ataques serão certamente sentidos em todo o mundo. Somados a ações igualmente reprováveis, de retirada da Organização Mundial da Saúde (OMS) e desmantelamento da Agência dos Estados Unidos para o Desenvolvimento Internacional (USAID, acrônimo em inglês) - ainda que pesem algumas ações dúbias desta última no passado - projetam um quadro de desesperança para a saúde pública global no futuro próximo. A ação conjunta de agências internacionais e do NIH foi fundamental para o enfrentamento da pandemia de COVID-19, por exemplo, como foi ao longo dos anos frente ao HIV/aids. Nada indica que novos desafios à saúde em escala mundial não se apresentarão nos anos vindouros, ainda mais tendo em vista o agravamento da crise climática - outra área de pesquisa, aliás, na alça de mira das equipes de demolição do governo Trump 2.

A comunidade científica mundial terá que procurar alternativas para lidar com o desastre em curso nos Estados Unidos, assim como denunciar os abusos e procurar apoiar como possível os cientistas atingidos pelos mesmos. O Brasil, em particular, deve procurar diversificar e fortalecer múltiplas parcerias, para minimizar os inevitáveis impactos negativos de um governo em franco enfrentamento com a ciência.

# Translation: Trump 2 en guerra contra la ciencia

**DOI:** 10.1590/0102-311XES027025

**Authors:** Kenneth Rochel de Camargo

**Affiliations:** 1 Instituto de Medicina Social Hesio Cordeiro, Universidade do Estado do Rio de Janeiro, Rio de Janeiro, Brasil.

El ascenso de Donald Trump al poder por segunda vez, apoyado en una alianza de autoritarismo, fundamentalismo religioso, supremacismo blanco y ultraneoliberalismo, ha sido visto con aprensión por la comunidad científica estadounidense e internacional desde que se anunciaron los resultados de la elección. El recuerdo reciente del negacionismo climático, la política errática frente a la pandemia de COVID-19, el extraño intento de alterar manualmente un mapa de pronóstico de trayectoria de un ciclón y otros actos similares en su primera administración justificaron este sentimiento de alarma. Y, como era de esperar, el ataque a la ciencia, en una escala aún mayor de lo esperado, es parte del verdadero *blitzkrieg* (guerra relámpago) que la “nueva-vieja” administración desató contra las instituciones y el marco legal de ese país. Revistas científicas de importancia inmediata, como *The Lancet* y el *British Medical Journal* (BMJ) publicaron editoriales que daban la alarma ^1^ ^,^ ^2^.

El ataque a la ciencia se produce en varios frentes. En primer lugar, la determinación de reducir la fuerza de trabajo al servicio del gobierno estadounidense, como parte de una política de recorte de gastos de cualquier manera y sin mayor criterio, afecta duramente a varios organismos relacionados con la investigación, como la Fundación Nacional de Ciencias (NSF, por su sigla en inglés) ^3^ ^,^ ^4^, que se ve obligada a reducir su personal en hasta un 50% en los próximos meses, además de tener su presupuesto amenazado con una reducción de hasta dos tercios del valor anterior ^4^. Amenazas similares pesan sobre otras agencias relevantes para la investigación, como la Administración Nacional Oceánica y Atmosférica (NOAA, por su sigla en inglés), organismo climatológico oficial del gobierno estadounidense, y la Administración Nacional de Aeronáutica y del Espacio (NASA, por su sigla en inglés).

Desde el punto de vista sanitario, la situación es aún más preocupante. Además de ser también afectado por la furia ciega de los recortes de gastos a toda costa, el nombramiento de Robert F. Kennedy Jr. como nuevo secretario del Departamento de Salud y Servicios Humanos (DHSS, por su sigla en inglés), equivalente al cargo de ministro de salud, es en sí mismo alarmante. RFK, como se le conoce, ha sido caracterizado repetidamente como un negacionista de la ciencia, en especial antivacunas, y forma parte de un grupo de doce individuos identificados como los mayores diseminadores de información antivacunas en internet ^5^. Un episodio en particular destaca: después de un grave error que causó la muerte de dos niños tras ser vacunados en Samoa (Polinesia), la interferencia de RFK exacerbó aún más la crisis de confianza en las vacunas, lo que llevó a una reducción aún mayor en la cobertura vacunal que llevó a la muerte de decenas de niños en la nación archipiélago del Pacífico ^6^. La nominación de RFK para este cargo ya había sido ampliamente denunciada, incluso por la Asociación Americana de Salud Pública (APHA, por su sigla en inglés), en una carta abierta publicada en su sitio *web* ^7^.

El DHSS abarca en su ámbito diversas instituciones esenciales para la investigación en salud, como el Instituto Nacional de Salud (NIH, por su sigla en inglés) y el Centro para el Control y la Prevención de Enfermedades (CDC, por su sigla en inglés). Una de las primeras medidas de Trump fue congelar cualquier actividad de estas agencias, interrumpiendo los comités que sugerirían propuestas de financiamiento, conferencias esenciales para la acción e investigación en salud, la contratación de personal e incluso la publicación científica ^8^ y la difusión de datos ^9^, interrumpiendo por primera vez en 60 años la publicación del *Morbidity and Mortality Weekly Report* (MMWR), importante informe epidemiológico ^1^. Esto se restableció unos 15 días después, pero aparentemente con importantes vacíos en el seguimiento del brote de gripe aviar que se desarrolla en ese país.

El candidato a la presidencia del NIH, Jay Bhattacharya, obtuvo notoriedad durante la pandemia de COVID-19 por ser uno de los signatarios de la *Declaración de Great Barrington*, un documento negacionista que criticaba las medidas de distanciamiento social desde una perspectiva economicista y que sirvió de base a propuestas absurdas de delegar el control de la epidemia a una supuesta inmunidad de rebaño en un momento en el que aún no había vacunas disponibles ^10^. El NIH es el mayor financiador de la investigación biomédica en Estados Unidos, uno de los mayores, si no el mayor, del mundo, y la interferencia ya prometida por la nueva administración en su dirección nubla las perspectivas futuras de desarrollo científico en este ámbito.

La determinación de recortar gastos en el NIH, además de reducir los fondos totales para el financiamiento directo de la investigación, compromete aún más su desarrollo al determinar un tope del 15% para los llamados costos indirectos, utilizados por las universidades e institutos de investigación para cubrir gastos con laboratorios, equipos y personal esenciales para la infraestructura de investigación, que estaban en un nivel promedio del 30%, y podían ser más del doble en el caso de las grandes universidades ^4^ ^,^ ^11^. 

Además de nombrar a personas claramente inadecuadas para cargos de liderazgo, restringir fondos y recortar personal, la actual administración también pretende censurar a los científicos prohibiendo la financiación de investigaciones y la publicación de artículos de funcionarios del gobierno que contengan cualquier referencia a palabras que hayan sido incluidas en un *index prohibitorum* digno de la Inquisición. Con base en la cruzada ideológica dirigida a las iniciativas de diversidad, equidad e inclusión (DEI), así como a cualquier referencia a la noción de género, en especial a la existencia misma de las personas transgénero, se eliminaron páginas enteras de sitios *web* gubernamentales, autores de artículos ya enviados para publicación exigieron su devolución a las revistas a las que los habían enviado, e incluso los fondos ya asignados tienen un futuro incierto ^8^ ^,^ ^12^.

Aunque las reacciones contrarias se han multiplicado en los últimos días, sobre todo en el ámbito judicial, en el que varios jueces han emitido fallos bloqueando algunos de los excesos más flagrantes de esta etapa inicial del nuevo gobierno, es un hecho que al menos durante los próximos dos años, hasta las próximas elecciones legislativas de medio término en Estados Unidos, Trump tiene el control de la Cámara, el Senado e incluso la Corte Suprema, lo que limita mucho el alcance de esa resistencia.

Las perspectivas para el desarrollo de la ciencia en Estados Unidos en los próximos años, en especial en el ámbito de la salud, son sombrías. Dado el papel relevante que desempeña esta nación en el escenario internacional de la ciencia, los efectos de estos ataques seguramente se sentirán en todo el mundo. A acciones igualmente reprobables se suman la retirada de la Organización Mundial de la Salud (OMS) y el desmantelamiento de la Agencia de los Estados Unidos para el Desarrollo Internacional (USAID, por su sigla en inglés) -a pesar de algunas acciones dudosas de esta última en el passado- proyecta un escenario desesperanzador para la salud pública mundial en el futuro cercano. La acción conjunta de agencias internacionales y del NIH fue fundamental para enfrentar la pandemia de COVID-19, por ejemplo, como lo fue a lo largo de los años frente al VIH/sida. No hay indicios de que en los próximos años no surjan nuevos desafíos para la salud a escala global, especialmente considerando el agravamiento de la crisis climática, otro ámbito de investigación, de hecho, en la mira de los equipos de demolición del gobierno Trump 2. 

La comunidad científica mundial tendrá que buscar alternativas para enfrentar el desastre en curso en Estados Unidos, así como denunciar los abusos y tratar de brindar el mayor apoyo posible a los científicos afectados por ellos. Brasil, en particular, debe buscar diversificar y fortalecer múltiples alianzas, para minimizar los inevitables impactos negativos de un gobierno en franca confrontación con la ciencia.
